# Healthcare cost and race: analysis of young women with stroke

**DOI:** 10.1186/s12939-023-01886-7

**Published:** 2023-04-21

**Authors:** Molly Jacobs, Charles Ellis

**Affiliations:** 1 Department of Health Services Research, Management and Policy, University of Florida, 1225 Center Drive, Gainesville, FL 32603 USA; 2 Department of Speech, Language, and Hearing Sciences, University of Florida, 1225 Center Drive, Gainesville, FL 32603 USA

**Keywords:** Stroke, Health disparities, Race, Health care costs, Young women

## Abstract

**Background:**

Over the last decade, the prevalence of young stroke has increased 40% particularly among vulnerable populations. These strokes are often more severe with worse outcomes. However, few studies have examined the impact on annual healthcare costs.

**Methods:**

Data from the 2008 to 2018 Medical Expenditure Panel Survey (MEPS) was used to identify a sample of female stroke survivors aged 18 and 60. MEPS includes demographics, health status, healthcare use, and expenditures for all participants providing the largest nationally representative data source of healthcare costs in the US. First, differences in racial and ethnic healthcare expenditure among young women with stroke were evaluated controlling for insurance type and demographic characteristics. Second, the relationship between healthcare expenditure and 1) time post stroke, 2) comorbidities, 3) healthcare utilization, and 4) post-stroke functional status was assessed. Finally, differential healthcare quality was tested as a potential mitigating differential.

**Results:**

Young Black women with stroke spend roughly 20% more on healthcare than White women after controlling for insurance, time post-stroke, healthcare utilization, and demographic differences. Costs remain 17% higher after controlling for comorbidities. Differences in expenditure are larger if survivors have diabetes, high blood pressure, or high cholesterol (78%, 24%, and 28%, respectively). Higher expenditure could not be explained by higher healthcare utilization, but lower quality of healthcare may explain part of the differential.

**Conclusion:**

Young Black women with stroke have 20% greater healthcare expenditure than other groups. Cost differentials cannot be explained by differentials in comorbidities, utilization, time post stroke, or functionality. Additional research is needed to explain these differences.

## Background

Trends of increasing stroke incidence at younger ages coupled with improvements in technology and treatment interventions reducing stroke fatalities have resulted in a larger population of people living post-stroke [28]. Depending on stroke severity, an individual experiencing a stroke at age 70 can expect to live between five- and 13-years post-stroke [32]. Those surviving a stroke often suffer from residual functional disabilities, emotional problems, and cognitive deficits [2, 27]. Advances in stroke care and the rising incidence of stroke at younger ages are expected to increase the lifetime cost of care from $36.7 to $94.3 billion between 2015 and 2035 [7, 34].

While evidence suggest that the prevalence of young stroke is increasing among individuals below age 65, stroke risk is not increasing uniformly among demographic groups [4]. Studies note higher rates of increase in the prevalence of young stroke among women compared to men citing variation in age-based risk factors such as taking oral contraception containing estrogen, experiencing gestational hypertension, or use of menopausal hormone therapy as the cause of the disparity [6]. Racial disparities in the incidence of first stroke among those age 20 to 54 increased from 26 to 48 among whites and 83 to 128 among blacks per 100,000 population between 1993 and 2005 [18]. The higher prevalence of stroke risk factors, including hypertension, diabetes mellitus, and smoking coupled with lower socioeconomic status, lower access to care, genetic predisposition, and limited awareness has contributed to these disparities [3, 17].

Despite the increasing cost of care and rising prevalence among young women, few studies have assessed the impact that these trends have on the individual cost of healthcare [1]. Evidence suggests that minority patients often face longer emergency department wait times, poor care quality, and biases in care delivery [4], but it remains unknown whether these inequities result in differential expenditures. Furthermore, young women, who do not meet age requirements for Medicare (a federal health insurance for people 65 and older), are often participating in the labor market and reliant on their employment for both income and health care coverage. Therefore, costly treatment and rehabilitation services can compound the economic burden if coupled with lost productivity. It follows that having a better understanding of the financial burden of post-stroke healthcare among patients below 60 could inform the burden of stroke faced by marginalized groups.

Using 10 years of data from the Medical Expenditure Panel Survey (MEPS), analysis quantifies disparities in the cost of healthcare by race/ethnicity, socioeconomic status, and household characteristics [25]. The role of comorbidities, post-stroke functional status, and healthcare utilization is enumerated while controlling for demographic variables, including age, employment, and regions [36].

## Methods

This study used the Medical Expenditure Panel Survey (MEPS), a household survey of U.S. noninstitutionalized populations. The Agency for Healthcare Research and Quality (AHRQ) has administered and maintained the MEPS since 1996. The MEPS collects information on variety of areas including demographics, health status, healthcare use, and expenditures of all participants. This analysis utilizes MEPS data collected between 2008 and 2018.

### Study population

This study included participants 18 years of age or older who provided a valid yes or no response to the question, “{Have/Has} {you/{PERSON}} ever been told by a doctor or other health professional that {you/he/she} had a stroke or TIA? A TIA is a transient ischemic attack which is sometimes referred to as a ministroke” during the study period. When a respondent reported having been diagnosed with stroke, the interviewer also asks about the date of diagnosis. Since we are unable to determine whether respondents who answered “I don’t know” had ever been diagnosed with stroke, we limited our sample to only individuals who answered yes regarding stroke diagnosis.

### Outcome measure

The main dependent variable is total annual healthcare expenditure which includes expenditures for eight types of medical events: hospital stays, emergency room visits, outpatient department visits, office-based medical provider visits, dental visits, home health care, other medical expenses, and prescription medicines. Expenditures include all direct payments to providers by individuals, private insurance (including TRICARE), Medicaid, and other public sources such as the Veterans Administration and Workers' Compensation. The MEPS expenditure data are based on household-reported information on health care use and expenditures supplemented with data obtained through a survey of providers.

In modeling consumer expenditure, a standard approach is to apply a natural log transformation to prices then fitting either a generalized linear or gamma regression model with a log-link function. Using the logarithmic transformation of healthcare expenditure, we evaluated the association between individual healthcare expenditure and insurance type, time post-stroke, demographic characteristics, and race/ethnicity using weighted least squares regression. Measures of healthcare utilizations, comorbidities, and functionality were iteratively added to the model to test the sensitivity and robustness. Finally, we tested for racial/ethnic differences in quality of care using logistic regression. Since quality of care is difficult to quantify and often requires subjective tools of measurement, two possible indicators of quality of care were used—having blood pressure checked by a health professional within the last two years and having a routine checkup within the last within the past 3 years.

### Covariates

Covariates included in the analysis were: age in years (18–85), family income (in logarithmic form), family size (1–14), year of survey completion, body mass index (BMI), education (years of post-primary schooling), and race/ethnicity (white, blacks, Hispanic and others). The “other” race category included those reporting being Asians, American Indians, native Hawaiian, and those reporting multiple races. Binary variables included an indicator for being a regular smoker, covered by Medicaid health insurance, married, and employed. An indicator for Medicaid was used since women aged 18 to 60 are not yet eligible for Medicare and, therefore, this was the second most common form of health insurance with private insurance being the most prevalent.

### Conditions

We considered a set of indicator variables for several health conditions that are known to affect health status. These included diabetes, hypertension, and high cholesterol. Participants were asked if they had ever been diagnosed with the condition (yes or no). The selection of these potential risk factors was based on prior literature [5, 10, 35].

### Functionality

To assess the association between healthcare cost and functional limitation, we used two survey questions. Limitations in physical activities are measured by asking, “{Do/Does} {you/{PERSON}} have difficulties walking, climbing stairs, grasping objects, reaching overhead, lifting, bending, or stooping, or standing for long periods of time?” Limitations in mental or cognitive functioning are assessed by asking, “{Do/Does} {you/{PERSON}} experience confusion or memory loss such that it interferes with daily activities?”.

### Healthcare utilization

Additionally, we considered a set of indicator variables for utilization of various types of health services. These included office-based, emergency room, outpatient, and inpatient visits as well as the total number of home health provider days and number of monthly prescription medications including refills. These comprise the primary forms of healthcare measured by MEPS and cover most major types of respondent healthcare utilization [30].

### Quality of care

Quality of care is highly correlated with health outcomes and healthcare utilization [33]. Prior research suggests that historically oppressed populations are significantly more like to receive lower quality healthcare than Whites [31]. This lower quality healthcare is associate with worse health outcomes in primary, acute, and post-acute care settings particularly among disadvantaged and marginalized groups [19]. While conventional wisdom suggests that lower quality of care will cost less, and higher quality care will cost more, only 34 percent of studies examining the relationship between quality and cost found a positive relationship while 30 and 36 percent reported a negative or negligible difference, respectively [14].

Despite mixed results, variation in quality of care received by racial and ethnic groups of young women post stroke could help to explain a portion of the variation in health care expenditure. To test this hypothesis, a measure of health care quality is needed. However, quality of care is often difficult to measure particularly in the absence of dedicated surveys about the patient and family experience [20]. Therefore, proxies are an important source of information given that most patients or survey respondents cannot be re-interviewed [26]. We use two previously tested instruments which have been shown to be highly correlated with quality of care [29]. Having a blood pressure check by a health professional within the last two years and having a routine checkup within the last within the past 3 years are used as general indicators of quality of healthcare. These items were drawn from the MEPS Self-Administered Questionnaire (SAQ), a paper-and-pencil questionnaire consisting of items from the Consumer Assessment of Healthcare Providers and Systems (CAHPS) and the Columbia Impairment Scale (CIS), designed to collect annual health status and health care quality and preventive health care measures of adults aged 18 and older in MEPS households. After 2018, these questions were only administered every other year.

### Statistical analysis

The MEPS survey has a complex survey design, which considers survey weights, strata, and clustering of individuals to provide nationally representative results of U.S. non-institutionalized populations. Therefore, all analyses, including standard errors, were adjusted for the design using SAS 9.4 and its survey procedures [16]. First, descriptive characteristics by race/ethnicity were calculated. Bivariate analyses were explored using Chi-squared tests with cluster and stratification parameters and a *p*-value of *p* < 0.05 was considered statistically significant. Second, weighted least squares (WLS) regression models were constructed with total healthcare expenditure (in logarithmic form) adjusting for potential confounders. Initially, only demographics (age, marital status), health behaviors (smoking status, BMI), and human capital (insurance, family size, income, educational attainment, employment) characteristics were included. A second model was estimated to include time post stroke. A third and fourth model included comorbidities (diabetes, high blood pressure, high cholesterol) and healthcare utilization (office visits, emergency room visits, inpatient visits, outpatient visits, home health visits), respectively. Finally, the last set of estimates included an indicator of physical limitations (difficulty walking).

## Results

### Descriptive statistics

The sample consists of longitudinal data for 1,597 (unweighted) young women (age < 60) with stroke. As seen in Table 1, the average age of the sample is 48 (sd = 9.7) with an average BMI of 30 and roughly a high school education. Women are roughly nine years (sd = 8.4) post stroke living in household between two and three people (sd = 1.6) with average household incomes $52,252 (sd = $1,274.33). Average annual healthcare expenditure by all payers is $14,297 (sd = $851.42) and average out of pocket expenditure is $1,058.57 (sd = $3451.16). Roughly half of women are married, and 32 percent are covered by Medicaid. Over half of women were employed and one-third smoke regularly. Sixty percent of young women had high blood pressure, half had been diagnosed with high cholesterol, and a quarter had diabetes. On average, healthcare utilization patterns reflected frequent usage: 11.1 (sd = 0.55) office-based provider, 0.8 (sd = 0.04) emergency department, 1.5 (sd = 0.16) outpatient, 0.4 (sd = 0.03) inpatient, and 12.0 (sd = 1.60) home health visits each year. While only 81 percent of the sample had a routine checkup within the 2 last years, 93 percent reported having their blood pressure checked.

**Table 1:** Descriptive statistics for key covariates related to cost of healthcare among young women with stroke

	Mean	Std Dev	Min	Max	Hispanic	White	Black	Other
	*N* = 1,597				*N* = 536	*N* = 601	*N* = 379	*N* = 81
Age	48.04	9.27	18	59	47.59	48.01	47.98	49.59
Age at Stroke	39.16	12.34	0	59	37.71	39.26	39.4	42.08
Years Post Stroke	8.78	8.43	-1	57	9.81	8.68	8.49	7.54
BMI	31.08	8.57	0.2	69.4	30.91	31.71	31.88	27.31
Years of education	12.52	3.71	0	17	10.65	12.51	12.02	12.39
Family Size	2.77	1.62	1	14	3.07	2.71	2.79	2.35
Family Income	52,252.07	44,442.92	-2150	346,617	41,548.7	43,556.91	30,260.33	38,257
Total Self Payment	1058.57	3451.16	0	103,015	1135.72	1183.34	671.21	575.87
Total Healthcare Expenditure	14,296.96	27,359.4	0	423,121	12,736.49	14,891.37	14,352.41	11,114.93
Office Visits	11.39	17.7	0	176	10.97	11.68	10.29	11.25
ER Visits	0.72	1.41	0	11	0.79	0.76	0.87	0.62
Outpatient Visits	1.36	6.13	0	140	1.47	1.82	1.23	0.73
Inpatient Visits	0.38	0.93	0	9	0.4	0.4	0.39	0.33
Home Health Visits	10.22	52.78	0	475	14.11	11.05	10.42	11.89
Smoker	31%				22%	36%	32%	36%
Medicaid	32%				38%	39%	49%	41%
Married	47%				45%	38%	20%	30%
Employed	54%				48%	46%	44%	58%
High BP DX	62%				56%	67%	77%	74%
High Cholesterol DX	50%				48%	54%	56%	41%
Diabetes DX	24%				25%	24%	30%	25%
Walking Limitations	43%				40%	46%	47%	43%
Doctor Check BP in last 2 years	93%				89%	94%	92%	94%
Routine Checkup in last year	81%				78%	83%	86%	74%

**Fig. 1:**
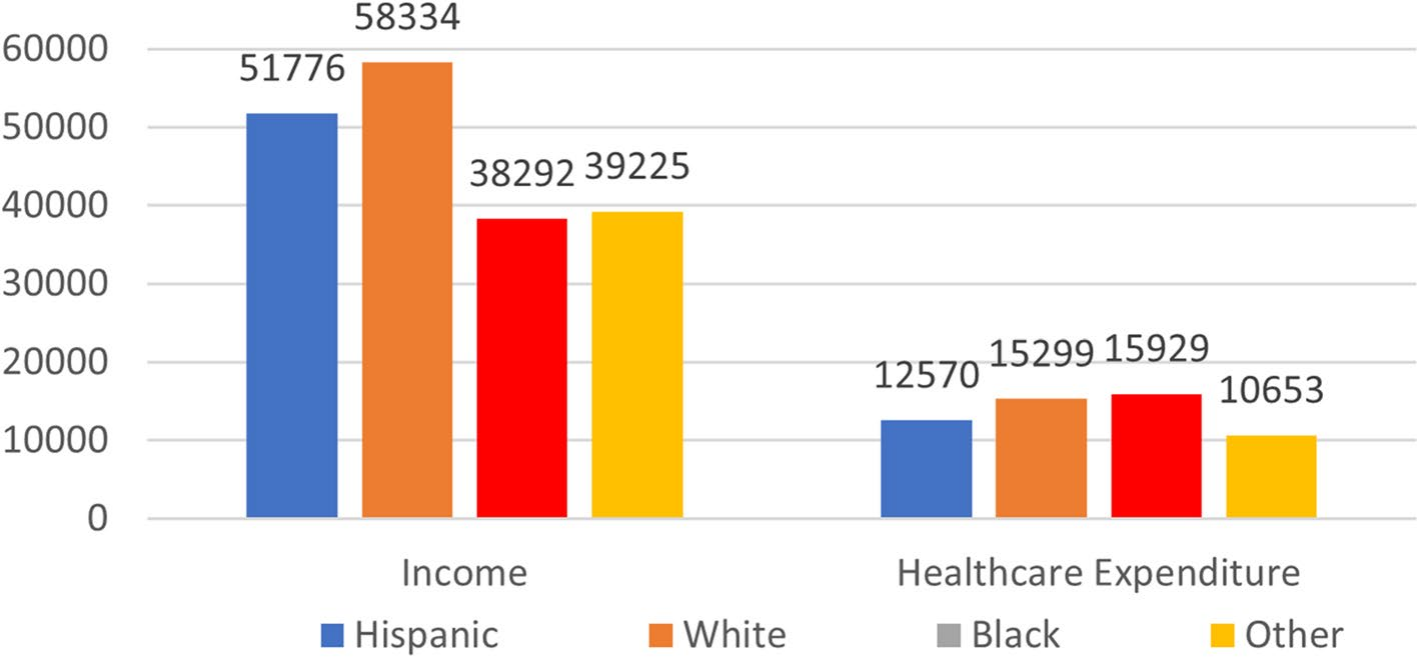
Income and Healthcare Expenditure by Race/Ethnicity

The sample is 30 percent Hispanic, 48.5 percent non-Hispanic White, 15 percent, non-Hispanic Black, and five percent other races. As seen in Fig. 1, Black women have lower income than women of other races, but the highest annual healthcare expenditure. Despite the high expenditure, Blacks do not have the highest utilization.

### Regression analysis

To better understand the relationship between expenditure and race, healthcare utilization, and prevalence of comorbidities, WLS regression analysis is run on all five sets of covariates described above. Results are listed in Table 2.

**Table 2:** Healthcare expenditure among young women with stroke: health, demographic, and utilization correlates

N	1096		1010		1010		1005		999	
R^2^	.10		.11		.17		.48		.49	
	Base Model (a)		Years Post Stroke (b)		Comorbidities (c)		Healthcare Utilization (d)		Physical Limitations (e)	
	β	Std Err	β	Std Err	β	Std Err	β	Std Err	β	Std Err
Intercept	-1.46	5.65	-2.53	8.67	11.67	7.99	**-49.51**	6.26	**-5.34**	6.21
Age	**.01**	.00	**.01**	.00	.00	.00	**.01**	.00	**.01**	.00
Smoker	**-.20**	.09	**-.23**	.10	**-.23**	.07	**-.19**	.07	**-.20**	.07
BMI	.05	.04	.03	.04	-.02	.03	**-.11**	.02	**-.12**	.02
Medicaid	**.62**	.07	**.66**	.07	**.51**	.09	**.18**	.06	**.16**	.07
Time	.00	.00	.00	.00	.00	.00	**.03**	.00	**.03**	.00
Family Size	**-.11**	.01	**-.10**	.02	**-.11**	.02	**-.04**	.01	**-.04**	.01
Family Income	**.20**	.02	**.21**	.02	**.25**	.02	**.17**	.01	**.16**	.01
Married	**.12**	.04	.08	.05	.03	.04	**.18**	.04	**.21**	.04
Years of Education	**.06**	.01	**.06**	.01	**.04**	.01	.00	.00	.00	.00
Employed	**-.73**	.08	**-.71**	.08	**-.57**	.07	**-.17**	.06	-.09	.06
Years Post Stroke			**-.02**	.00	**-.01**	.00	**-.01**	.00	**-.01**	.00
Diabetes DX					**.84**	.06	**.49**	.06	**.48**	.06
High BP DX					**.27**	.11	.08	.11	.08	.10
High Cholesterol DX					**.28**	.04	**.23**	.02	**.23**	.02
Office Visits							**.03**	.00	**.03**	.00
ER Visits							**.17**	.01	**.16**	.01
Outpatient Visits							**.05**	.00	**.04**	.00
Inpatient Visits							**.56**	.01	**.57**	.01
Home Health Visits							**.01**	.00	**.01**	.00
Walking Limitations									**.27**	.03
Hispanic	.12	.08	.13	.09	.14	.10	.07	.07	.04	.07
Black	**.20**	.07	**.22**	.08	**.13**	.10	**.16**	.07	**.17**	.06
Other Race	**.25**	.07	**.22**	.08	**.20**	.10	.11	.07	.11	.08

Dependent Variable: Total Healthcare Expenditure

Reference Group: Non-Hispanic White

**Indicates** Significant at 95% Confidence Level

Data Source: Medical Expenditure Panel Survey 2008–2018

Estimates are weighted using longitudinal sampling weights

- *Base model*: As expected, healthcare expenditure increases significantly with age by roughly one percent per year. It is also higher for individuals who are married, have higher educational attainment, and income. Individuals with more financial resources can spend more on their health and are able to purchase additional preventative health services. Healthcare expenditure is lower for those in larger families suggesting that family members might provide care informally in the home thus alleviating the need to pay for outside services. Women who are employed spend significantly less than those who are not employed since they are likely healthier and require fewer healthcare services. Smokers spend less each year on healthcare. This result likely reflects a lower level of concern for their level of physical health which is reflected in their smoking behavior.
- *Years post stroke*: Results show little change when the number of years post-stroke is added to the model. Healthcare expenditure appears to decline roughly two percent with each additional year after the stroke even for women under 60 suggesting that they have likely completed acute and post-stroke rehabilitation care. Even after controlling for time post stroke, Blacks still have significantly higher healthcare expenditure than other racial groups.
- *Comorbidities*: Diagnosis with diabetes, high blood pressure, and high cholesterol increases healthcare expenditure by 84 percent, 27 percent, and 28 percent respectively. Not surprisingly, women with diabetes had significantly higher expenditures than those with other comorbidities given the numerous costly medications often required to control diabetes and the cost of managing long term diabetes related complications. Relative healthcare expenditure for Blacks remains significantly higher despite accounting for the impact of comorbidities on healthcare expenditure.
- *Healthcare utilization*: Parameters denoting the level of annual healthcare utilization are positively related to healthcare expenditure since high levels of utilization increase costs. Each additional office, emergency, outpatient, inpatient, and home healthcare visit increases annual healthcare expenditure by 3 percent, 17 percent, 5 percent, 56 percent, and 1 percent respectively. Even after the additional of comorbidity and healthcare utilization covariates, racial, human capital, and demographic characteristics remain statistically significant.
- *Physical limitations*: Women who report difficulty walking post-stroke, not surprisingly have healthcare expenditure 27 percent higher than those without impaired mobility.

### Quality of Care

To evaluate racial and ethnic differences in quality of care received by young women post-stroke, logistic regression compared the likelihood of having had a blood pressure check by a health professional within the last two years and having had a routine checkup within the last within the past 3 years—two metrics which instrument for care quality—between racial and the ethnic groups controlling for demographics (age, marital), health behaviors (smoking status, BMI), and human capital (insurance, family size, income, educational attainment, employment). Results listed in Table 3 indicate no racial/ethnic differences in the likelihood of blood pressure screening and suggest that Blacks have a higher likelihood (OR = 1.604, CI = 0.751, 3.449) of receiving a routine checkup while other racial groups have a lower likelihood (OR = 0.489, CI = 0.193, 0.842). BMI, age, marital status, and years of education appear to be more highly correlated with these quality-of-care indicators.

**Table 3:** Indicators of quality of healthcare: blood pressure and routine checkups

	Routine Check in the Last Year					Blood Pressure Checked in Last 2 Years				
N	873					831				
	β	Std Err	OR	95% CI		β	Std Err	OR	95% CI	
Intercept	-52.08	126.80				-210.50	205.20			
Age	**.03**	.01	1.03	1.01	1.06	.02	.02	1.02	.98	1.06
Hispanic	-.11	.21	.79	.44	1.44	.01	.37	.83	.28	2.46
Black	**.59**	.26	1.60	2.91	3.45	.02	.31	.84	.29	2.50
Other Race	**-.60**	.30	.49	.19	0.84	-.22	.52	.66	.14	3.14
Smoker	-.64	.36	.53	.26	1.08	-.79	.46	.46	.18	1.13
BMI	.66	.59	1.94	.60	6.27	**1.53**	.58	4.62	1.47	14.54
Medicaid	.13	.16	1.30	.69	2.44	.16	.17	1.38	.71	2.66
Year	.02	.06	1.02	.90	1.16	.10	.10	1.11	.91	1.36
Family Size	.01	.10	1.01	.83	1.22	-.06	.10	.94	.78	1.15
Family Income	.14	.13	1.15	.88	1.51	-.26	.36	.77	.38	1.57
Married	-.25	.39	.78	.36	1.69	**.85**	.42	2.34	1.01	5.41
Years of Education	**.08**	.04	1.08	1.01	1.16	**.13**	.04	1.13	1.05	1.23
Employed	-.57	.33	.56	.29	1.09	-.36	.57	.70	.22	2.18
Dependent Variable: Routine checkup in the last year						Dependent Variable: Blood pressure check in the last 2 years				
Reference Group: Non-Hispanic White						Reference Group: Non-Hispanic White				
**Indicates** Significant at 95% Confidence Level						**Indicates** Significant at 95% Confidence Level				
Data Source: Medical Expenditure Panel Survey 2008–2018						Data Source: Medical Expenditure Panel Survey 2008–2018				
Estimates are weight using longitudinal sampling weights						Estimates are weight using longitudinal sampling weights				

## Discussion

Using a nationally representative sample of young women with stroke, this study showed that annual healthcare expenditure was higher for Black women and women of other historically oppressed racial groups than Whites. Results were robust to inclusion of demographic, healthcare utilization, comorbidity, and physical limitation controls. Black women had between 15 and 20 percent greater annual healthcare expenditures than White women with the same frequency of care and confounding conditions. These results are troubling given the lower average income (Black: $30,260.33, sd = 33,605.75; White: $43,556.91, sd = 51,323.89) and education attainment (Black: 12.0, sd = 3.7; White: 12.5, sd = 2.8) of Black women compared to White. These disparities in healthcare expenditure could neither be explained by variability in the time post stroke, nor differential patterns of healthcare utilization. While racial difference in the quality of healthcare could explain a portion of the differences, variation is quality was difficult to estimate using MEPS data. While some studies have used other covariates as “instruments” for healthcare quality, only one of those instruments used here (receiving routine care within the last three years) showed a statistically significant difference between demographic subgroups.

To the authors knowledge, this is the first study examining racial disparities in healthcare expenditure among young women with stroke. Husaini and co-authors (2013) examined racial differences in healthcare costs using an age-inclusive, mixed gender cohort of stroke survivors. Using data from 2008, their results showed that treatment costs associated with stroke were higher among Blacks than Whites ($41,370 versus $30,215, *P* < 0.001). The expenditure differential persisted when they compared average annual healthcare costs for the entire year of 2008 and remained intact when the comparisons were made simply for stroke cost ($74,338 for blacks versus $55,884 for whites, *P* < 0.001). While their analysis did not specifically focus on women, they observed similar trends of Black males ($74,006 versus $59,403, *P* < 0.001) and Black females ($74,589 versus $52,877, *P* < 0.001) compared to Whites—a differential that they attributed to higher comorbidity prevalence and longer hospitalizations for Blacks. While information on stroke type was not available for the present study, other researchers have noted persistence of racial disparities among both ischemic and hemorrhagic stroke [13].

While age was not a criterion used in their sample, Yu, et al. noted gender related cost differentials using a sample of Canadian men and women [37]. Their findings showed that in the year prior to stroke, women had higher unadjusted healthcare costs compared to men and this differential persisted in the year following stroke onset. They suggested that moderate and severe stroke (versus mild stroke), intracerebral hemorrhage (versus ischemia), and higher baseline frailty (versus lower frailty) were associated with increased cost, while rural residence was associated with lower cost compared to those living in non-rural areas. While the analysis presented here indicated that utilization did not mitigate disparities in healthcare expenditure, the authors noted that acute care accounted for most healthcare care during the first-year post-stroke while home care and long-term care comprised healthcare costs in later years [37].

While variation in the quality of care received was one possible explanation for the higher healthcare expenditure that we examined, variation in the severity of comorbidities could also have contributed. Additionally, disparities in the timing when Black women with chronic conditions sought medical services could lead to higher cost of care if they obtained care later in the disease progression than Whites [12]. Finally, the higher cost among Black females may exist because, as suggested by previous studies, they are more likely to discontinue behavioral and pharmacological therapies which can, in turn, lead to more complications [22].

However, determination of which factor(s) contribute to racial disparities in post-stroke healthcare expenditure is outside the scope of this analysis. Nevertheless, the expenditure differentials identified are significant given that the costs and lifelong morbidity associated with stroke are great when stroke occurs in the young [9]. This information is important for several reasons. First, the cost stroke care and the distribution of the economic burden of stroke is necessary to inform cost-effectiveness studies of treatments and interventions. Second, identifying racial differences in cost may highlight areas of inequity or generate opportunities to reduce costs, which are relevant for policy makers and health system planning.

## Limitations

Despite the importance of the findings presented here, this study and the data used in this analysis face several limitations. First, MEPS contains no information on the type of stroke or stroke severity. Furthermore, both strokes and TIAs are grouped together as a single survey item, but prior research has shown racial differences in TIAs similar to stroke [11, 15, 21]. Subsequently, the sample likely contains both individuals with little or no stroke-related impairments as well as individual with severe or debilitating post-stroke conditions. While efforts were made to account for this variability in the analysis, the data contains no indicator of stroke impact.

Second, MEPS does not contain any information on the total number of strokes or stroke-like event experienced by respondents. The duration post stroke is calculated as the difference between the respondent current age and the age at which they reported experiencing a stroke. However, we are unable to determine if this duration is measured from the first ever or most recent stroke. Since we cannot determine how individuals reported their age at stroke, we are unable to guarantee that our interpretation of this covariate reflects accurately reflects respondent reporting intention.

Third, the authors of this study hypothesize that racial disparities in healthcare expenditure could be related to differences in the quality of healthcare received by women of different racial and ethnic groups. MEPS incorporates the Consumer Assessment of Healthcare Providers and Systems (CAHPS®) into the self-administered questionnaire (SAQ) to measure quality of care from the consumer’s perspective. However, CAHPS® only refers to events which occurred over the last 12 months that involve immediate or specific types of care. Additionally, these CAHPS® questions elicit subjective responses making it difficult to compare between individuals who might have different standards of care. Instead, this study elected to use more objective metrics, but these measures are imperfect.

Finally, like with many other federally administered surveys, race/ethnicity data in MEPS is limited with regards to completeness and accuracy and subject to recall bias. While race/ethnicity data is available, race/ethnicity data are problematic for Asian Americans and Pacific Islanders (AAPIs), and for American Indians and Alaskan Natives (AIANs). These groups are collapsed into a single category, along with multiracial respondents, labeled as “Other Race.” As a result, incomplete and inaccurate race/ethnicity data limit our understanding of the sources of disparities in health care cost, quality, and outcomes. Because of these limitations, analyses using race/ethnicity data from MEPS are generally restricted to the identification of differences between Blacks and Whites.

## Conclusion

Even though stroke is the third leading cause of death in women, relatively few studies focus exclusively on women with stroke and even fewer among young women with stroke [24]. This study examined healthcare expenditure among young women with stroke and identified significant racial disparities. Identification and characterization of these cost disparities was the goal of the current study, but additional research is needed to fully explain cost differentials [8]. Since both the quality of care and facility play a role in the cost of care, these factors should be potential targets for projects designed to improve outcomes and to decrease the risk of stroke racial minorities. While the data used in this analysis precluded the examination of the severity of illness as well as postoperative complications, these can be critical determinates of the cost of healthcare [23]. To explore these and other factors related to the cost of stroke, additional research is needed.

## Data Availability

This study utilizes the Medical Expenditure Panel Survey (MEPS). Lynn A. Blewett, Julia A. Rivera Drew, Risa Griffin and Kari C.W. Williams. IPUMS Health Surveys: Medical Expenditure Panel Survey, Version 1.1 [dataset]. Minneapolis, MN: IPUMS, 2019. https://doi.org/10.18128/D071.V1.1**.** Data can be found at https://www.meps.ahrq.gov/mepsweb/data_stats/download_data_files.jsp.
